# Supplementary material for: Ambient heat exposure and COPD hospitalisations in England: a nationwide case-crossover study during 2007–2018
Source: Thorax. 2022 Apr 22;77(11):1098–104. doi: 10.1136/thoraxjnl-2021-218374 (PMC9606528; doi:10.1136/thoraxjnl-2021-218374)
Supplement: Supplementary data [file thoraxjnl-2021-218374supp001.pdf]

Online Supplement: "Ambient heat exposure and COPD hospitalisations in England: A nationwide case-crossover study during 2007-2018."

Garyfallos Konstantinoudis[1,*], Cosetta Minelli[2], Ana Maria Vicedo Cabrera[3,4], Joan Ballester[5], Antonio Gasparrini[6,7,8], and Marta Blangiardo[1]

[1] *MRC Centre for Environment and Health, Department of Epidemiology and Biostatistics, School of Public Health, Imperial College London, London, UK*

[2] *National Heart and Lung Institute, Imperial College London, London, United Kingdom*

[3] *Institute of Social and Preventive Medicine, University of Bern, Switzerland*

[4] *Oeschger Center for Climate Change Research, University of Bern, Bern, Switzerland*

[5] *ISGlobal, Barcelona, Spain.*

[6] *Department of Public Health Environments and Society, London School of Hygiene Tropical Medicine, London, UK*

[7] *Centre for Statistical Methodology, London School of Hygiene Tropical Medicine, London, UK*

[8] *Centre on Climate Change and Planetary Health, London School of Hygiene Tropical Medicine, London, UK*

[*] Corresponding author. Email: g.konstantinoudis@imperial.ac.uk

Konstantinoudis G, *et al. Thorax* 2022; 77:1098–1104. doi: 10.1136/thoraxjnl-2021-218374

Contents

S1 Text **4**

S1.1 Modelling relative humidity 4

S1.1.1 Model 4

S1.1.2 Cross-validation 5

S1.1.3 Results 5

S1.2 Statistical analysis 7

S1.2.1 WAIC analysis 7

S1.2.2 Age-sex effect modification 7

S1.2.3 Spatial effect modification 8

S1.3 Confounders/ Mediators/ Effect modifiers 10

List of Tables

S1 Mean, standard deviation (sd), median, interquartile range (IQR), min and max of maximum summer temperature [^{o}C] across England during 2007-2018. 12

S2 Median and interquartile range (IQR) of the temperature [^{o}C], daily mean $PM_{2.5}$ [$\mu g/m^3$], daily mean of the 8 hours of maximum O_3 [$\mu g/m^3$] and relative humidity [%] across event, non-event days and the different lags preceding the hospitalisation event. 13

S3 Mean, standard deviation (sd), median, interquartile range (IQR), min and max of daily mean $PM_{2.5}$ [$\mu g/m^3$] exposure across England during summers 2007-2018. 14

S4 Mean, standard deviation (sd), median, interquartile range (IQR), min and max of daily mean of the 8 hours of maximum O_3 [$\mu g/m^3$] exposure across England during summers 2007-2018. 15

S5 Mean, standard deviation (sd), median, interquartile range (IQR), min and max of daily median of relative humidity [%] exposure across England during summers 2007-2018. 16

S6 Percentage hospitalisation risk of COPD for every 1°C increase in summer temperature using the model adjusted for relative humidity and national holidays and the different temperature thresholds c. 17

S7 Median and 95% credible intervals of the % risk COPD hospitalisation for every 1° increase in warm temperatures by age group and sex. RH+NL refers to adjustment for relative humidity and national holidays, whereas RH+NL+POL for additional adjustment for $PM_{2.5}$ and O_3. 18

List of Figures

S1 Boundaries of the 326 Lower Tier Local Authorities of England in 2015 (median size: 208km^2). . . 19

S2 The spatial distribution of the index of multiple deprivation using quintiles in 2015 in England at the lower tier local authority level. Q1 indicates the most deprived areas. 20

S3 The spatial distribution of urbanicity in based on the Office for National Statistics classification in 2011 at the lower tier local authority level. 21

S4 The quintiles of the spatial distribution of the proportion of a lower tier local authority that is covered by green land such as woodland, agricultural land, grassland and other natural vegetated land as classified in the Land Cover Map 2015. 22

S5 The spatial distribution of the average temperature [^oC] by lower tier local authority during 2007-2018 in England. 23

S6 Relative hospitalisation risk (relative to the risk at 18^oC) using 3rd degree of b-splines at 3 knots and the model with total age and sex and adjusted for national holidays and relative humidity. The red dashed line indicated the threshold c used throughout the study. 24

Konstantinoudis G, *et al. Thorax* 2022; 77:1098–1104. doi: 10.1136/thoraxjnl-2021-218374

S1 Text

S1.1 Modelling relative humidity

S1.1.1 Model

Data on relative humidity in England during 1862-2019 is available nationwide from MetOffice through the HadUK-Grid product (`https://catalogue.ceda.ac.uk/`). The spatial resolution of HadUK-Grid can vary from 1km×1km to 60km×60km, nevertheless the highest temporal resolution available are months. MetOffice also provides daily data on relative humidity during 1853-2019 for each meteorological station through the Met Office Integrated Data Archive System (MIDAS) product (`https://catalogue.ceda.ac.uk/`). To retrieve daily relative humidity data nationwide and not only at the meteorological stations, we employ the following modelling framework:

Let $Y_{jkt}(s)$ be the arcsin transformation of the relative humidity from MIDAS and $X_{kt}(s)$ the nationwide relative humidity from HadUK-Grid in location s, day j, month k and year t:

$$\begin{aligned}
Y_{jkt}(s) &\sim \text{Normal}\big(\mu_{jkt}(s), \sigma_1\big) \\
\mu_{jkt}(s) &= \beta_0 + bX_{kt}(s) + \gamma_j + \omega_t + u(s) \\
\gamma_j &\sim \text{AR1}(\sigma_2, \rho) \\
\omega_t &\sim \text{Normal}(0, \sigma_3^2) \\
u(s) &\sim \text{GMRF}(\sigma_4, \phi) \\
\sigma_1, \sigma_2, \sigma_3, \phi, \rho &\sim \text{PCpriors} \\
\beta_0, b &\sim N(0, \delta)
\end{aligned} \tag{1}$$

wheres $\mu_{jkt}(s)$ and σ_1 be the variables of the normal distribution, β_0 and b the regression coefficients, γ_j a random effect capturing the daily temporal trends, ω_t a random effect capturing the yearly trends, $u(s)$ the spatial autocorrelation term (based on the Stochastic Partial Differential Equation Approach [1]) and $\sigma_2, \sigma_3, \sigma_4, \rho,$ and ϕ the corresponding variance and correlation hyperparameters.

The specification of the PCpriors of the hyperparameter is the following: for σ_1 we specify that the probability of observing arcsin relative humidity larger than 10 is 0.10. Similarly for σ_2 and σ_3, we specify that the probability of observing arcsin relative humidity larger than 1 due to the temporal trends is 0.10. For the correlation parameter ρ of the autoregressive process of order 1, we select a probability of 0.5 for correlations of 0.5 reflecting our lack of knowledge with respect to the correlation structure in the data. For the Gauss Markov Random Field

Konstantinoudis G, *et al. Thorax* 2022; 77:1098–1104. doi: 10.1136/thoraxjnl-2021-218374

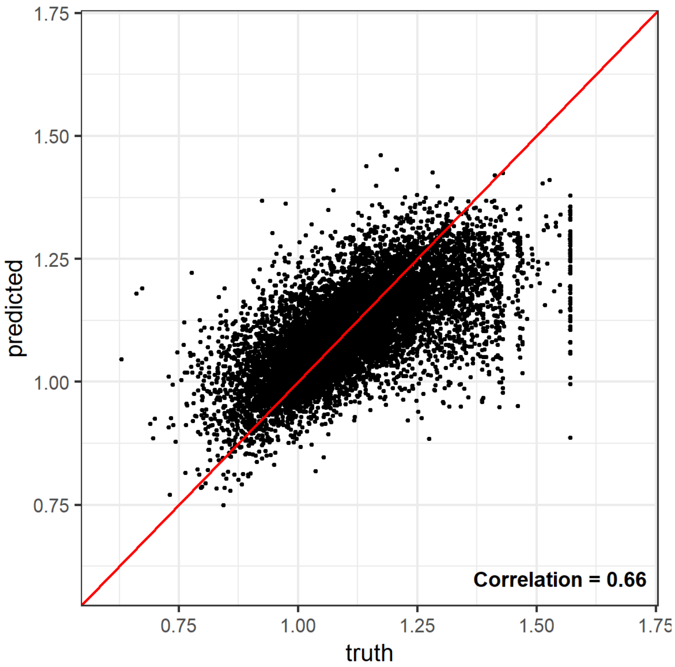

Figure 1.1. Predicted versus true values of the arcsin relative humidity for a sample of 10,000 values in the summer months during 2007-2018 in England.

(GMRF) term we select the standard deviation hyperparameter σ_4 as in the temporal case, whereas for the range parameter ϕ we select ranges larger that 10km with probability of 0.5. For more information about the PCpriors and their mathematical formulation see [2, 3]. Lastly, δ was fixed to 0.001 for the intercept, whereas to 0.1 for b.

S1.1.2 Cross-validation

We performed the following leave one out cross validation scheme: Let N be the total number of meteorological stations during 2007-2019, first we divided N randomly by 10 groups, and for each (out of the 10) step we excluded the entire time series of the group of the randomly sampled N/10 meteorological stations. Figure 1.1 shows the results of the cross validation, and in particular a scatterplot between the observed and predicted values. The correlation between truth and predicted is relatively high, ie 0.66, indicating that our model have good predictive ability.

S1.1.3 Results

Table 1.1 shows the results of Model 1, Figure 1.3 shows the mean of the daily median of relative humidity in 2013 for the 3 summer months. The maps show that areas around London had lower relative humidity during the summer months in 2013. The relative humidity seems to be consistently higher in South West during the

Table 1.1. Mean, standard deviation, median and 94% credible intervals of the intercept, the covariate and the hyperparameters of Model 1.

Random variables	mean	sd	median	2.5%	97.5%
β_0	0.144	0.009	0.144	0.126	0.161
b	0.012	0.000	0.012	0.012	0.012
$1/\sigma_1^2$	101	0.285	101	101	102
$1/\sigma_2^2$	172	8.30	172.26	156.30	189
ρ	0.386	0.028	0.384	0.332	0.440
$1/\sigma_3^2$	68.1	26.3	63.8	29.8	131
$1/\sigma_4^2$	0.063	0.007	0.063	0.052	0.079
ϕ	8,384	1,557	8,352	5,493	11,600

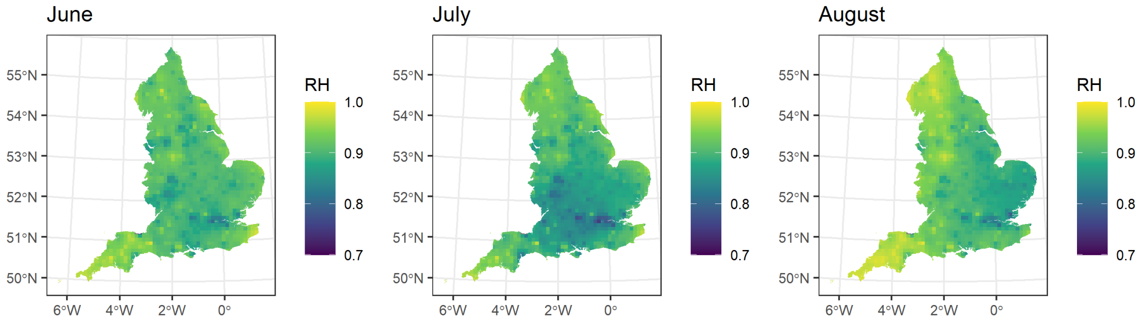

Figure 1.3. Maps of mean of median relative humidity by summer month in 2013.

summer 2013.

Konstantinoudis G, *et al. Thorax* 2022; 77:1098–1104. doi: 10.1136/thoraxjnl-2021-218374

S1.2 Statistical analysis

In this subsection we will introduce the mathematical notation of the models used in the main analysis.

S1.2.1 WAIC analysis

Let Y_{tjk} be the case-control identifier for the chronic obstructive pulmonary disease (COPD) hospitalisation for the event (case or control) at time t, in the j-th case-control group and k-th patient. Let also X_1t be the temperature at t event Z_{1t}, Z_{2t}) a vector denoting the different confounders (relative humidity and holiday) at the t-th time point. Then:

$$\begin{aligned}
Y_{tjk} &\sim \text{Poisson}(\mu_{itjk}) \\
\log(\mu_{tjk}) &= \alpha_1 I(X_{1t} < c_l)X_{1t} + \alpha_2 I(X_{1t} \geq c_l)X_{1t} + \sum_{m=1}^{2} \beta_m Z_{mt} + u_j + w_k \\
u_j &\sim N(0, 100) \\
w_k &\sim N(0, \sigma_1^2) \\
a_1, a_2, \beta_1, \ldots \beta_4 &\sim N(0, 1) \\
\sigma_1 &\sim \text{Gamma}(1, 2)
\end{aligned}$$

In the above equation, a_1 is the effect of temperatures lower than the threshold c, a_2 is the effect of temperatures higher or equal than the threshold c, $I(\cdot)$ an indicator function, β_1, β_2 the effects of the confounding, u_j a fixed effect on the j-th case control group and w_k a random effect to account for recurrent hospitalisations. The normal distributions read N(mean, variance). We ran the above model for the different temperature thresholds c_l for $l = 1, 2, \ldots, 10$ representing the 50-th, 55-th, ... 95-th percentiles of the temperature and computed the WAIC [4]. Removing the term $\sum_{m=1}^{2} \beta_m Z_{mi}$ and the corresponding priors of β_1, β_2 results in the unadjusted models.

S1.2.2 Age-sex effect modification

Let c_* be the temperature threshold that minimises the WAIC from Step 1. Expanding the indices of the above model results in the models for the age and sex effect modification. Let g be the age-sex index representing individuals aged $0 - 64$, $65 - 74$ and > 75 years old or the total group and males, females or the total group.

Konstantinoudis G, *et al. Thorax* 2022; 77:1098–1104. doi: 10.1136/thoraxjnl-2021-218374

The above model can be rewritten as follows:

$$\begin{aligned} Y_{tjkg} &\sim \text{Poisson}(\mu_{tjkg}) \\ \log(\mu_{tjkg}) &= \alpha_1 I(X_{1tg} < c_*) X_{1tg} + \alpha_2 I(X_{1tg} \geq c_*) X_{1tg} + \sum_{m=1}^{2} \beta_m Z_{mtg} + u_j + w_k \\ u_j &\sim N(0, 100) \\ w_k &\sim N(0, \sigma_1^2) \\ a_1, a_2, \beta_1, \dots \beta_5 &\sim N(0, 1) \\ \sigma_1 &\sim \text{Gamma}(1, 2) \end{aligned}$$

S1.2.3 Spatial effect modification

On the third step of the analysis we let the coefficient of the temperature higher than c_* vary by lower tier local authorities (LTLA). Let $H_{1i}, \dots, H_{8i})$ be the spatial effect modifiers representing the green space, the quintiles of deprivation, the urbanicity categories and the average temperature in the s-th LTLA. We can write:

$$\begin{aligned} Y_{tjk} &\sim \text{Poisson}(\mu_{tjk}) \\ \log(\mu_{tjk}) &= \alpha_1 I(X_{1t} < c_*) X_{1t} + \alpha_{2s} I(X_{1t} \geq c_*) X_{1t} + \sum_{m=1}^{2} \beta_m Z_{mt} + u_j + w_k \\ \alpha_{2s} &= \alpha_2 + \sum_{q=1}^{8} \gamma_q H_{sq} + v_s + b_s \\ w_k &\sim N(0, \sigma_1^2) \\ v_s &\sim N(0, \sigma_2^2) \\ b_s | b_{-s} &\sim N\left(\frac{\sum_{s \sim r} w_{rs} b_s}{\sum_{s \sim r} w_{rs}}, \frac{\sigma_3^2}{\sum_{s \sim r} w_{rs}} \right) \\ u_j &\sim N(0, 100) \\ a_1, \beta_1, \dots \beta_4, \gamma_1, \dots, \gamma_8 &\sim N(0, 1) \\ a_2 &\sim N(0.0425, 0.0039^2) \\ \sigma_1, \sigma_2, \sigma_3 &\sim \text{Gamma}(1, 2). \end{aligned}$$

w_{rs} are neighborhood weights and are 1 when the r and s LTLAs are neighboring (we write $s \sim r$) and 0 otherwise, $\gamma_1, \dots, \gamma_8$ are the effects of the spatial effect modifiers and the $v_s + b_s$ the BYM prior [5]. Unstructured overdispersion is captured on v_s and spatial autocorrelation on b_s. The hyperparameters σ_2^2, σ_3^2 are the variance

parameters of the unstructured and structured random effects. Removing the term $\sum_{q=1}^{8} \gamma_q H_{sq}$ and the corresponding priors of $\gamma_1, \ldots, \gamma_8$ results in the model without the adjustment for spatial effect modifiers, while allowing the effect of warm temperatures to vary in space.

Konstantinoudis G, *et al. Thorax* 2022; 77:1098–1104. doi: 10.1136/thoraxjnl-2021-218374

S1.3 Confounders/ Mediators/ Effect modifiers

Directed acyclic graph for the relationship between temperature and hospitalisations for chronic obstructive pulmonary disease (COPD).

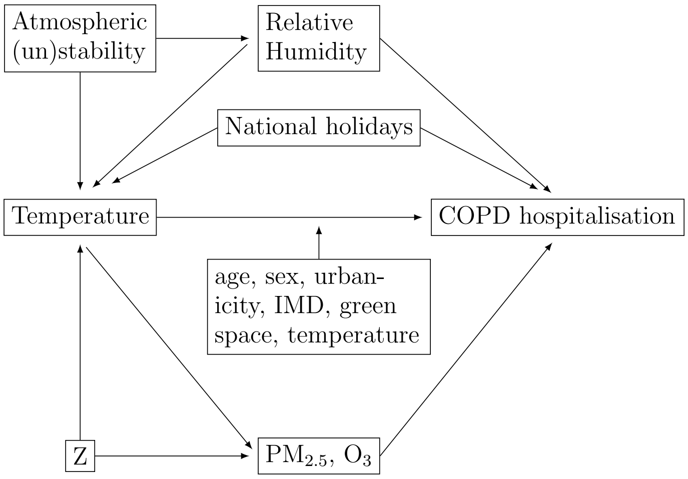

- **Relative humidity**: Previous studies has reported an association between relative humidity and COPD hospitalisations [6]. Relative humidity and temperature are both affected by factors such as atmospheric (un)stability and climate dynamics. Nevertheless, (soil and air) humidity determines the fraction of radiation (coming from the Sun and absorbed mainly by the surface) that is transformed into latent and sensible heat. Latent heat is generated due to phase transition of water, e.g., evaporation. The remaining radiation is transformed into sensible heat, leading to temperature changes [7]. Thus, relative humidity is likely a confounder.

- **National holidays**: can affect individual behaviours with respect to seeking health care services but also through other behaviours that can affect the temperature. Thus, holidays can be a potential confounder [8].

- **Air-pollution**: Previous studies have reported an association between short term exposure to $PM_{2.5}$ and O_3, and COPD hospitalisations [9, 10]. Thus these air-pollutants are expected to be correlated with COPD hospitalisations. Although temperature and air-pollutants have their own causal factors, e.g. air pollution emissions, they are also likely to have a shared cause Z, an example could be the already mentioned atmospheric (un)stability and climate dynamics, and temperature to affect $PM_{2.5}$ and O_3 concentration [11]. Thus, $PM_{2.5}$ and O_3 are likely to be mediators.

- **Effect modifiers**: The effect of temperature on COPD hospitalisations can be modified by, among other

Konstantinoudis G, *et al. Thorax* 2022; 77:1098–1104. doi: 10.1136/thoraxjnl-2021-218374

factors, age, sex, urbanicity, green space and average temperature. We selected these effect modifiers based on 1. consistency with the literature [8] and 2. Clear hypotheses about the mediation: We included age as the elderly have been reported to be more vulnerable, sex as differences can arise due to different lifestyle, occupational or biological factors, averaged temperature to account for potential adaptation to higher temperatures, urbanicity, to examine if urban heat island modifies the effect of temperature, and green space. Green space may reduce health risks in urban populations by removing air pollution, reducing noise, cooling temperature, enhancing physical activities, reducing psychological stress, and interaction with a clean environment [12]. 3. As the main interest of the current analysis was spatial effect modification, we did not include factors that vary significantly in time, such as air-pollution.

Konstantinoudis G, *et al. Thorax* 2022; 77:1098–1104. doi: 10.1136/thoraxjnl-2021-218374

Table S1: Mean, standard deviation (sd), median, interquartile range (IQR), min and max of maximum summer temperature [^{o}C] across England during 2007-2018.

year	mean	sd	median	IQR	min	max
2007	19.39	2.71	19.42	3.31	4.00	30.31
2008	19.63	2.90	19.39	3.47	6.35	30.20
2009	20.26	3.28	20.27	4.17	2.25	31.97
2010	20.49	3.25	20.38	4.28	6.41	32.83
2011	19.38	3.10	19.15	3.98	5.32	33.41
2012	19.05	3.42	18.89	4.27	2.83	33.05
2013	21.10	3.78	20.88	5.37	5.76	34.09
2014	20.63	3.16	20.53	4.33	6.38	32.30
2015	19.87	3.37	19.79	4.23	3.03	36.67
2016	20.46	3.36	20.31	4.09	5.26	34.21
2017	20.37	3.45	20.17	4.09	5.49	34.48
2018	22.42	3.98	22.20	5.86	4.98	35.66

Konstantinoudis G, *et al. Thorax* 2022; 77:1098–1104. doi: 10.1136/thoraxjnl-2021-218374

Table S2: Median and interquartile range (IQR) of the temperature [^{o}C], daily mean $PM_{2.5}$ [$\mu g/m^3$], daily mean of the 8 hours of maximum O_3 [$\mu g/m^3$] and relative humidity [%] across event, non-event days and the different lags preceding the hospitalisation event.

		Event days		Non-event days	
Covariate	lag	Median	IQR	Median	IQR
Temperature	0	20.91	4.17	20.93	4.13
	1	20.97	4.21	20.94	4.13
	2	20.92	4.23	20.90	4.15
	0-2	20.93	3.73	20.92	3.67
$PM_{2.5}$	0	9.23	5.60	9.10	5.45
	1	9.25	5.57	9.07	5.38
	2	9.23	5.44	9.06	5.25
	0-2	9.24	4.54	9.08	4.38
O_3	0	65.5	22.37	65.00	21.91
	1	66.08	22.68	65.37	21.82
	2	66.21	22.35	65.58	21.78
	0-2	65.94	19.45	65.31	18.81
Relative humidity	0	0.90	0.11	0.90	0.11
	1	0.90	0.11	0.90	0.11
	2	0.90	0.11	0.90	0.11
	0-2	0.90	0.08	0.90	0.08

Konstantinoudis G, *et al. Thorax* 2022; 77:1098–1104. doi: 10.1136/thoraxjnl-2021-218374

Table S3: Mean, standard deviation (sd), median, interquartile range (IQR), min and max of daily mean $PM_{2.5}$ $[\mu g/m^3]$ exposure across England during summers 2007-2018.

year	mean	sd	median	IQR	min	max
2007	8.71	5.51	7.47	5.13	0.10	76.97
2008	8.71	6.07	7.37	4.17	0.00	69.92
2009	8.81	4.55	7.48	4.14	1.15	62.24
2010	9.19	5.03	7.97	4.78	0.70	52.42
2011	9.20	4.50	8.03	4.41	0.87	71.39
2012	8.28	4.97	7.04	4.78	0.35	84.33
2013	10.42	7.12	8.20	8.26	0.00	75.41
2014	8.58	4.83	7.39	5.43	0.31	53.73
2015	7.86	4.88	6.75	5.79	0.03	44.60
2016	8.60	8.20	6.07	6.51	0.00	73.65
2017	8.05	6.04	6.20	6.20	0.00	65.88
2018	8.67	5.67	7.13	6.07	0.00	52.24

Konstantinoudis G, *et al. Thorax* 2022; 77:1098–1104. doi: 10.1136/thoraxjnl-2021-218374

Table S4: Mean, standard deviation (sd), median, interquartile range (IQR), min and max of daily mean of the 8 hours of maximum O_3 [$\mu g/m^3$] exposure across England during summers 2007-2018.

year	mean	sd	median	IQR	min	max
2007	70.15	18.75	67.33	18.59	5.99	198.68
2008	71.32	18.91	69.35	21.86	0.19	166.95
2009	69.10	20.51	64.09	22.77	3.09	180.97
2010	68.11	19.87	64.57	23.99	12.07	157.44
2011	67.02	16.06	65.18	18.66	7.75	145.45
2012	63.28	17.56	62.02	19.81	0.19	182.54
2013	71.60	19.99	69.04	23.01	0.00	186.98
2014	71.28	15.92	69.64	19.41	6.91	150.06
2015	71.69	17.33	69.33	21.35	2.09	177.66
2016	64.46	17.90	60.38	20.44	0.86	162.83
2017	63.17	17.04	60.39	18.88	12.31	246.06
2018	71.78	22.37	67.24	32.10	16.28	268.69

Konstantinoudis G, *et al. Thorax* 2022; 77:1098–1104. doi: 10.1136/thoraxjnl-2021-218374

Table S5: Mean, standard deviation (sd), median, interquartile range (IQR), min and max of daily median of relative humidity [%] exposure across England during summers 2007-2018.

year	mean	sd	median	IQR	min	max
2007	0.94	0.05	0.95	0.08	0.49	1.00
2008	0.93	0.06	0.95	0.09	0.51	1.00
2009	0.93	0.06	0.94	0.09	0.43	1.00
2010	0.91	0.07	0.93	0.12	0.46	1.00
2011	0.92	0.06	0.93	0.09	0.50	1.00
2012	0.96	0.05	0.97	0.06	0.54	1.00
2013	0.91	0.07	0.92	0.10	0.45	1.00
2014	0.91	0.06	0.91	0.09	0.50	1.00
2015	0.90	0.07	0.91	0.11	0.47	1.00
2016	0.94	0.06	0.95	0.08	0.50	1.00
2017	0.93	0.06	0.94	0.08	0.49	1.00
2018	0.88	0.09	0.90	0.14	0.37	1.00

Konstantinoudis G, *et al. Thorax* 2022; 77:1098–1104. doi: 10.1136/thoraxjnl-2021-218374

Table S6: Percentage hospitalisation risk of COPD for every 1°C increase in summer temperature using the model adjusted for relative humidity and national holidays and the different temperature thresholds c.

quantile	threshold c (^{o}C)	WAIC	Effect bellow c	Effect above c
50	20.6	2,257,389	0.12 (-0.22, 0.46)	1.56 (1.26, 1.84)
55	21.0	2,257,373	0.12 (-0.23, 0.48)	1.56 (1.26, 1.86)
60	21.3	2,257,371	0.16 (-0.21, 0.49)	1.53 (1.24, 1.81)
65	21.7	2,257,379	0.21 (-0.12, 0.57)	1.50 (1.21, 1.78)
70	22.1	2,257,333	0.22 (0.10, 0.55)	1.49 (1.22, 1.77)
75	22.6	2,257,331	0.37 (0.05, 0.65)	1.42 (1.15, 1.68)
80	23.2	2,257,273	0.37 (0.09, 0.65)	1.46 (1.19, 1.71)
85	23.8	2,257,330	0.44 (0.18, 0.70)	1.46 (1.20, 1.72)
90	24.9	2,257,440	0.62 (0.38, 0.86)	1.39 (1.11, 1.66)
95	26.5	2,257,351	0.72 (0.49, 0.93)	1.50 (1.20, 1.82)

Konstantinoudis G, *et al. Thorax* 2022; 77:1098–1104. doi: 10.1136/thoraxjnl-2021-218374

Table S7: Median and 95% credible intervals of the % risk COPD hospitalisation for every 1° increase in warm temperatures by age group and sex. RH+NL refers to adjustment for relative humidity and national holidays, whereas RH+NL+POL for additional adjustment for $PM_{2.5}$ and O_3.

Sex	Age group	Unadjusted models	RH+NL	RH+NL+POL
Males	0-64	0.97 (0.24, 1.73)	1.32 (0.57, 2.06)	0.64 (-0.24, 1.49)
Females	0-64	0.92 (0.25, 1.63)	1.14 (0.39, 1.84)	-0.04 (-0.90, 0.84)
Total	0-64	1.00 (0.51, 1.48)	1.28 (0.75, 1.82)	0.35 (-0.26, 0.98)
Males	65-74	1.13 (0.50, 1.71)	1.39 (0.74, 2.03)	0.62 (-0.15, 1.39)
Females	65-74	1.56 (0.94, 2.20)	1.75 (1.13, 2.41)	0.76 (0.01, 1.52)
Total	65-74	1.31 (0.83, 1.70)	1.54 (1.07, 2.03)	0.66 (0.13, 1.22)
Males	>75	1.41 (0.87, 1.91)	1.51 (0.98, 2.07)	0.25 (-0.35, 0.92)
Females	>75	1.29 (0.79, 1.83)	1.51 (0.94, 2.06)	0.47 (-0.14, 1.11)
Total	>75	1.36 (0.96, 1.71)	1.49 (1.11, 1.89)	0.38 (-0.10, 0.84)
Males	Total	1.23 (0.89, 1.59)	1.45 (1.10, 1.83)	0.51 (0.07, 0.94)
Females	Total	1.26 (0.93, 1.62)	1.46 (1.09, 1.82)	0.41 (-0.03, 0.84)
Total	Total	1.24 (0.98, 1.51)	1.47 (1.19, 1.73)	0.47 (0.16, 0.75)

Konstantinoudis G, *et al. Thorax* 2022; 77:1098–1104. doi: 10.1136/thoraxjnl-2021-218374

Figure S1: Boundaries of the 326 Lower Tier Local Authorities of England in 2015 (median size: 208km^2).

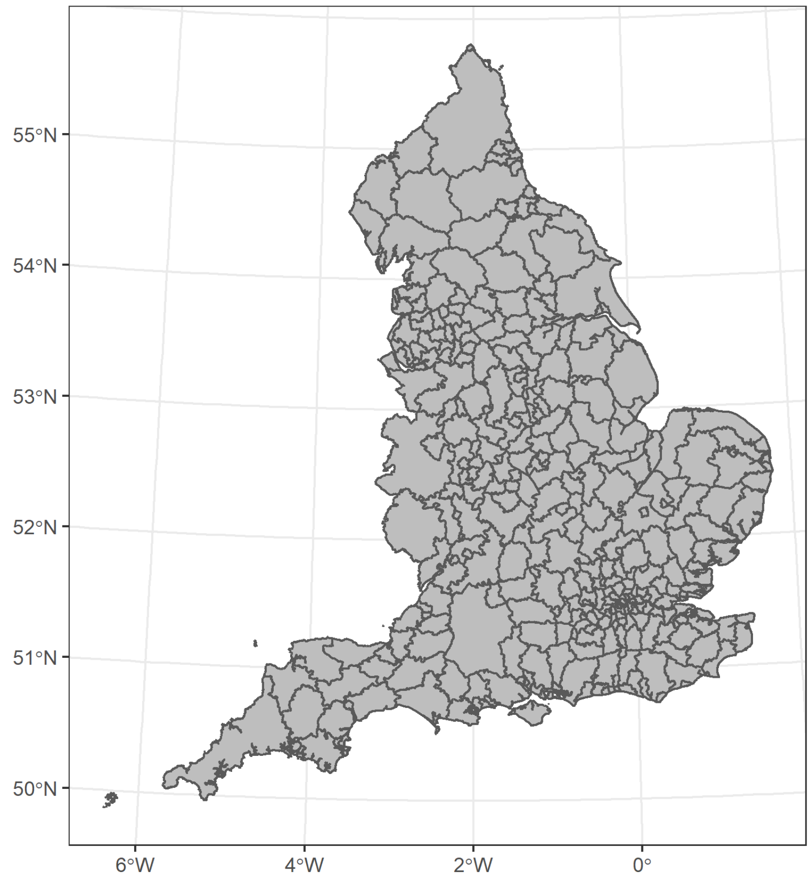

Konstantinoudis G, *et al. Thorax* 2022; 77:1098–1104. doi: 10.1136/thoraxjnl-2021-218374

Figure S2: The spatial distribution of the index of multiple deprivation using quintiles in 2015 in England at the lower tier local authority level. Q1 indicates the most deprived areas.

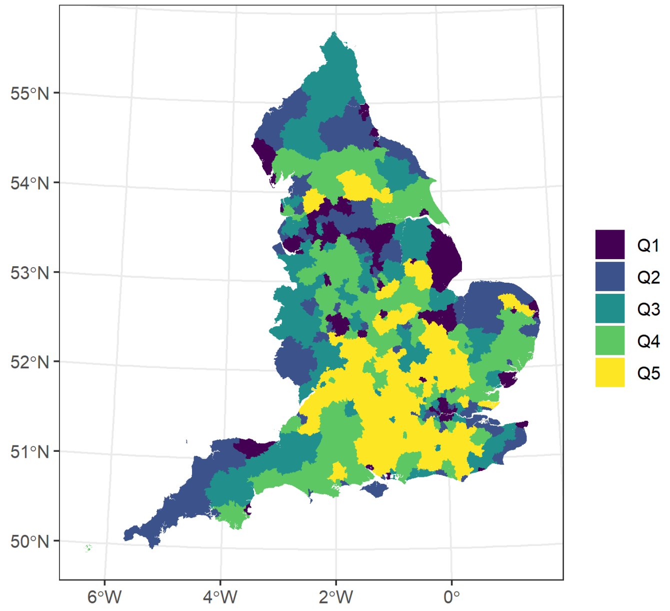

Konstantinoudis G, *et al. Thorax* 2022; 77:1098–1104. doi: 10.1136/thoraxjnl-2021-218374

Figure S3: The spatial distribution of urbanicity in based on the Office for National Statistics classification in 2011 at the lower tier local authority level.

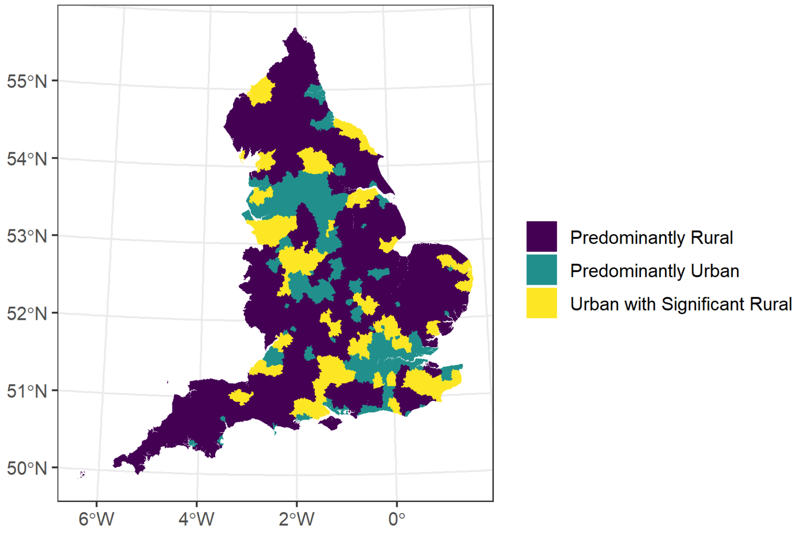

Konstantinoudis G, *et al. Thorax* 2022; 77:1098–1104. doi: 10.1136/thoraxjnl-2021-218374

Figure S4: The quintiles of the spatial distribution of the proportion of a lower tier local authority that is covered by green land such as woodland, agricultural land, grassland and other natural vegetated land as classified in the Land Cover Map 2015.

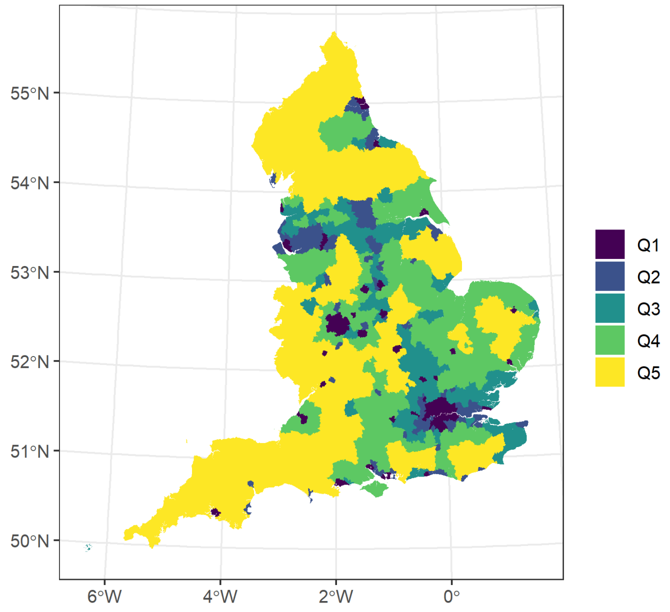

Konstantinoudis G, *et al. Thorax* 2022; 77:1098–1104. doi: 10.1136/thoraxjnl-2021-218374

Figure S5: The spatial distribution of the average temperature [^oC] by lower tier local authority during 2007-2018 in England.

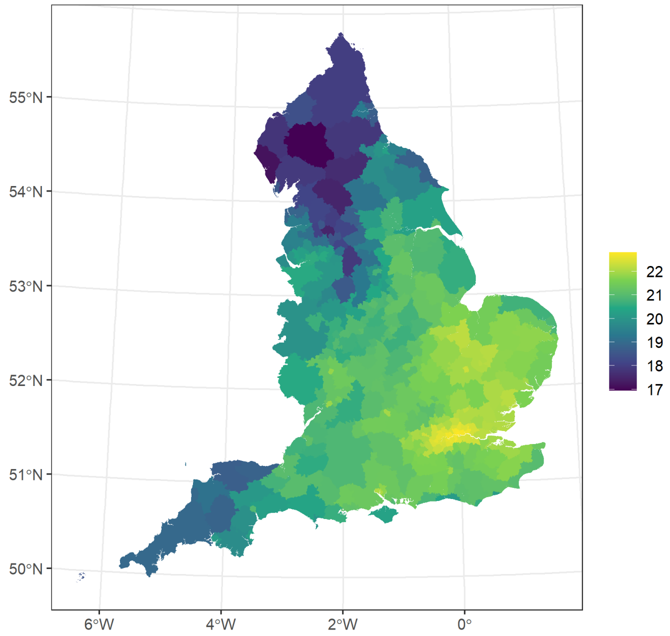

Konstantinoudis G, *et al. Thorax* 2022; 77:1098–1104. doi: 10.1136/thoraxjnl-2021-218374

Figure S6: Relative hospitalisation risk (relative to the risk at 18^oC) using 3rd degree of b-splines at 3 knots and the model with total age and sex and adjusted for national holidays and relative humidity. The red dashed line indicated the threshold c used throughout the study.

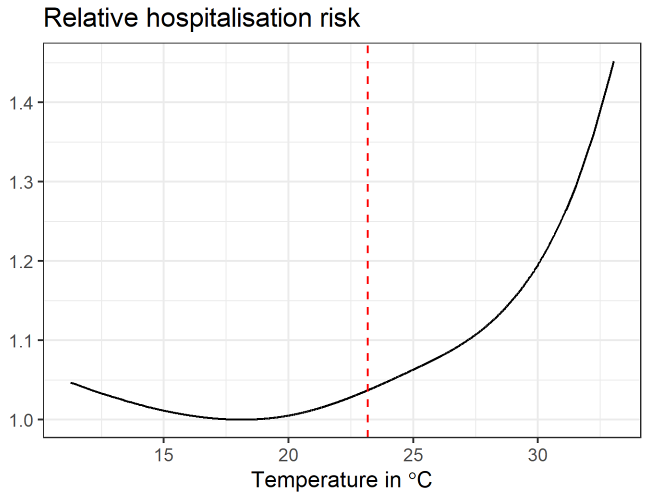

References

[1] Finn Lindgren, Håvard Rue, and Johan Lindström. An explicit link between gaussian fields and gaussian markov random fields: the stochastic partial differential equation approach. *Journal of the Royal Statistical Society: Series B (Statistical Methodology)*, 73(4):423–498, 2011.

[2] Sigrunn Holbek Sørbye and Håvard Rue. Penalised complexity priors for stationary autoregressive processes. *Journal of Time Series Analysis*, 38(6):923–935, 2017.

[3] Daniel Simpson, Håvard Rue, Andrea Riebler, Thiago G Martins, and Sigrunn H Sørbye. Penalising model component complexity: A principled, practical approach to constructing priors. *Statistical science*, pages 1–28, 2017.

[4] Andrew Gelman, Jessica Hwang, and Aki Vehtari. Understanding predictive information criteria for bayesian models. *Statistics and computing*, 24(6):997–1016, 2014.

[5] Julian Besag, Jeremy York, and Annie Mollié. Bayesian image restoration, with two applications in spatial statistics. *Annals of the institute of statistical mathematics*, 43(1):1–20, 1991.

[6] Jovan Javorac, Marija Jevtić, Dejan Živanović, Miroslav Ilić, Sanja Bijelović, and Nataša Dragić. What are the effects of meteorological factors on exacerbations of chronic obstructive pulmonary disease? *Atmosphere*, 12(4), 2021.

[7] Erich M Fischer, Sonia I Seneviratne, Pier Luigi Vidale, Daniel Lüthi, and Christoph Schär. Soil moisture–atmosphere interactions during the 2003 european summer heat wave. *Journal of Climate*, 20(20):5081–5099, 2007.

[8] James E Bennett, Marta Blangiardo, Daniela Fecht, Paul Elliott, and Majid Ezzati. Vulnerability to the mortality effects of warm temperature in the districts of england and wales. *Nature Climate Change*, 4(4):269–273, 2014.

[9] Man-Hui Li, Li-Chao Fan, Bei Mao, Jia-Wei Yang, Augustine MK Choi, Wei-Jun Cao, and Jin-Fu Xu. Short-term exposure to ambient fine particulate matter increases hospitalizations and mortality in copd: a systematic review and meta-analysis. *Chest*, 149(2):447–458, 2016.

[10] Hui Gao, Kan Wang, William W Au, Wensui Zhao, and Zhao-lin Xia. A systematic review and meta-analysis of short-term ambient ozone exposure and copd hospitalizations. *International journal of environmental research and public health*, 17(6):2130, 2020.

[11] Jessie P Buckley, Jonathan M Samet, and David B Richardson. Commentary: Does air pollution confound studies of temperature? *Epidemiology*, 25(2):242–245, 2014.

[12] Iana Markevych, Julia Schoierer, Terry Hartig, Alexandra Chudnovsky, Perry Hystad, Angel M Dzhambov, Sjerp De Vries, Margarita Triguero-Mas, Michael Brauer, Mark J Nieuwenhuijsen, et al. Exploring pathways linking greenspace to health: Theoretical and methodological guidance. *Environmental research*, 158:301–317, 2017.
